# Does Cardiorespiratory Fitness Predict the Physiological and Psychological Stress Response to a Mathematics Exam in Secondary High School Students?

**DOI:** 10.1002/ejsc.70172

**Published:** 2026-04-08

**Authors:** Markus Gerber, Michelle Haller, Vera Nina Looser, Sebastian Ludyga

**Affiliations:** ^1^ Department of Sport Exercise and Health University of Basel Basel Switzerland

**Keywords:** coping, fitness, heart rate variability, reactivity, school, stress

## Abstract

School is widely recognized as one of the primary sources of stress among adolescents. While some studies employing laboratory‐based stressors have suggested that adolescents with better cardiorespiratory fitness (CRF) may exhibit lower stress reactivity to psychosocial stressors, research based on real‐life stressors is lacking. Therefore, we examined whether CRF predicts physiological and psychological reactivity in response to a real‐life stressor (mathematics exam). Students were recruited from Swiss public schools (9th grade). The final sample included 67 students (58% female, Mage = 15.09 years). Heart rate (HR), heart rate variability (HRV), mood states, and state anxiety were used as indicators stress reactivity. CRF was assessed using the 20m shuttle‐run test. Statistical analyses used regression analyses, which were controlled for relevant social and demographic confounders, as well as baseline outcomes during a nonstress condition (normal mathematics lesson). Exposure to the mathematics exam resulted in decreased HRV and mood, alongside increased state anxiety. While better CRF was associated with lower HR, higher HRV, better mood, and lower state anxiety across stress and baseline conditions, CRF did not predict physiological and psychological outcomes after controlling for baseline scores and confounders. Hence, our study suggests that although better CRF is associated with favorable physiological and psychological states, this relationship appears independent of students' current stress exposure. Further research employing other ecologically valid stressors is needed to better understand the impact of CRF on real‐life stress reactivity. From a school health perspective, it is essential to support students in developing the capacity to cope effectively with academic stressors.

## Highlights

- Exposure to a mathematics exam resulted in increased HR, decreased HRV, poorer mood, alongside increased anxiety.
- Better CRF was associated with lower heart rate and higher HRV across the baseline and stress condition.
- Better CRF was associated with better mood and lower state anxiety across the baseline and stress condition.
- The association between CRF and physiological and psychological states was independent of students' current stress exposure.

## 1 Introduction

A dysregulated stress response can have detrimental effects on adolescents' health. Based on the cognitive–transactional stress model, stress responses can vary considerably between individuals because they depend on multiple appraisal processes that are shaped by protective and vulnerability factors (Lazarus 1999). Persistently high stress levels can negatively affect the immune system (Glaser and Kiecolt‐Glaser 2005), decrease cognitive function (Ludyga 2017), and increase the risk of cardiovascular disease (Kivimaki and Steptoe 2018).

A survey conducted by the American Psychological Association showed that adolescents aged 15–17 perceive more stress than adults in many domains such as work, money, economy, or health‐related concerns (American Psychological Association 2018). During adolescence, many biological, psychological, and social changes occur, requiring constant adaptation. From a neurobiological perspective, adolescence is a sensitive period due to the maturation of certain brain regions. During adolescence, subcortical regions such as the amygdala and ventral striatum—central to emotion processing and reward sensitivity—undergo heightened reactivity, whereas the prefrontal cortex, which supports impulse control and emotion regulation, is still maturing. This developmental imbalance between affective and regulatory systems may increase emotional volatility and risk‐taking, thereby heightening vulnerability to mental disorders (Andersen and Teicher 2008). This vulnerability is particularly pronounced among adolescent girls, who exhibit a higher prevalence of stress‐related mental disorders, such as depression and anxiety, compared to their same‐aged male peers (Hampel and Petermann 2006).

Given that modern societies increasingly emphasize education and that young people's future prospects strongly depend on their academic performance, school is considered one of the most prominent sources of stress among adolescents. Consequently, the pressures of performance‐oriented societies may place excessive demands on young individuals (Högberg 2021). If adolescents do not develop effective strategies to manage stress, it can result in mental health problems (Pascoe et al. 2020). In this context, a prospective study from England with almost 5000 participants showed that academic stress at age 15 is associated with an increased risk of depression and self‐harm in young adulthood (Guo et al. 2026).

Researchers have proposed that people with good cardiorespiratory fitness (CRF) are better equipped to cope with stress. According to the American College of Sports Medicine, CRF is a component of physical fitness that reflects the capacity of the cardiopulmonary system (heart, lungs, and circulation) to deliver oxygen to working skeletal muscles to support sustained physical activity (ACSM 2025). The existence of such a stress‐buffering effect is well documented in children (Gerber, Endes, et al. 2017a) and adolescents (Haugland et al. 2003), and is commonly explained by the cross‐stressor adaptation (CSA) hypothesis. According to this hypothesis, repeated exposure to physical stimuli (e.g., endurance or strength training) induces both specific and nonspecific physiological adaptations. Thus, when a person repeatedly engages in physical activity, the body becomes more adept at managing physical stress (e.g., as experienced during high‐intensity training), due to a physiological learning effect, resulting in a blunted stress response. This represents a specific adaptation. In contrast, nonspecific adaptation refers to improvements that generalize to other stressors, including psychosocial ones. In line with the CSA hypothesis, Hamer et al. (2006) showed that a single (acute) exercise session was associated with a lower blood pressure response during a subsequent stressor task. Additionally, a meta‐analysis demonstrated that people with better fitness show an attenuated cardiovascular stress reactivity and faster recovery following exposure to psychosocial stressors (Forcier et al. 2006). These studies utilized a variety of stressor tasks. A review focusing specifically on the Trier Social Stress Test (TSST), one of the most widely used psychosocial laboratory stressors, found that seven out of 14 studies supported the notion that increased fitness can attenuate the response to psychosocial stress (e.g., lower cortisol levels, lower heart rate, higher heart rate variability). Two of the 14 studies also showed that participants with better fitness reported less state anxiety and felt calmer after completion of the TSST (Mücke et al. 2018). However, children and adolescents were underrepresented in the studies included in the review, as most samples consisted of young and middle‐aged adults, with only two studies (*N* = 368 participants, 49% male, age range: 8–13 years) specifically focusing on children and adolescents.

One key criticism of this line of research is that the external validity of laboratory‐based studies is limited. In other words, can the findings from the laboratory (where little or nothing is at stake for the participants) be generalized to real life? Although some studies suggest that better fitness may also confer protection against real‐life stress (von Haaren et al. 2016; Wyss et al. 2016), this field remains at its early stages, particularly with respect to adolescent populations. Against this background, the aim of the current study was to examine whether an academic real‐life stressor (exam in mathematics) would result in acute physiological and psychological stress reactions, and whether CRF predicts physiological and psychological reactivity in response to the mathematics exam, after controlling for nonstress baseline scores (assessed during a regular mathematics lesson) and potential confounding factors. Based on the literature presented above, we hypothesized that a real‐life academic stressor would trigger substantial stress reactions (Guo et al. 2026; von Haaren et al. 2016), and that better CRF would be associated with a more beneficial physiological and psychological stress reactivity (Mücke et al. 2018; von Haaren et al. 2016).

## 2 Materials and Methods

### 2.1 Study Design

An experimental (noninterventional) within–between interaction design was applied to examine whether better CRF would be associated with a more favorable physiological and psychological stress reactivity during a stress condition (mathematics exam), after controlling for relevant confounders and baseline scores assessed during a nonstress condition (regular mathematics lesson). Importantly, both conditions were not imposed by the researchers, as they were part of the existing curriculum.

### 2.2 Participants and Procedures

The procedures of this study were approved by the responsible ethical review board prior to data collection (EKNZ, 2022‐01438). Students were recruited from two public (secondary) schools from so‐called A‐track classes, which are characterized by high academic performance. Both schools were located in the northwestern, German‐speaking part of Switzerland. The Swiss school system is highly selective, with only around 20% of all Swiss students currently attending an academic high school, which provides access to a Swiss university. To gain admission to an academic high school, A‐track students must meet a minimum average grade requirement across multiple school subjects, with mathematics being among the most heavily weighted subjects.

**FIGURE 1:**
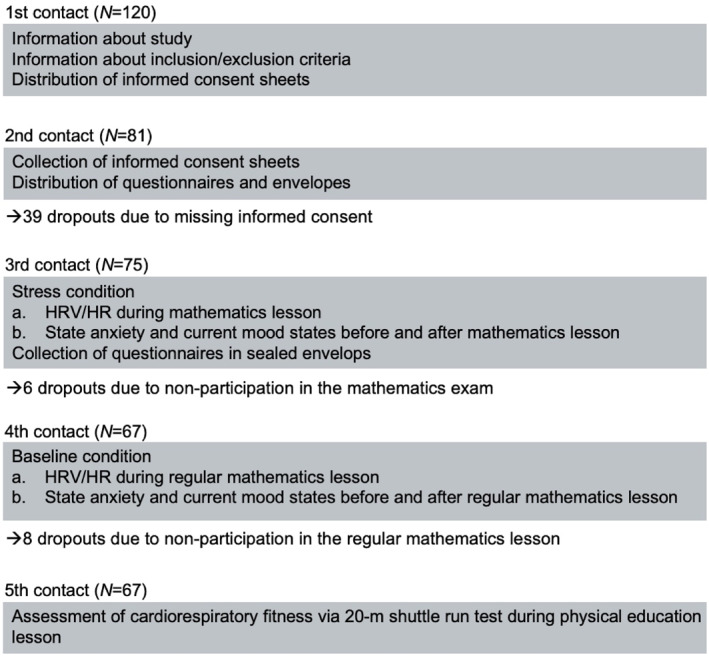
Study design and participant flow chart.

Schools were contacted via the school principals. Classes were selected randomly from a list of classes provided by the class teachers. Student information sessions and data collection were conducted during regular class time. Overall, the students and the investigator met on five occasions (see Figure 1 for an overview): During the first meeting, the investigator explained the objectives of the study and the procedures, and students had the opportunity to ask questions. The investigator then distributed the informed consent forms, which had to be signed by the adolescents and their parents/legal guardians. During the second meeting (after 1 week), students received a paper‐ and‐pencil questionnaire assessing their socio‐demographic background and psychological factors, which they were instructed to return in a sealed envelope by the next meeting. During the third meeting, students' stress reactivity was measured during the stress condition (mathematics exam). Each student was provided with a HR monitor and chest belt at the beginning of the first lesson of the day, which they were instructed to put on immediately. HR and HRV were recorded continuously until the end of the last morning lesson. At both the beginning and end of the mathematics lesson, the students completed a short questionnaire assessing their current (state) anxiety levels and mood states. The mathematics exam was scheduled during the second, third, or fourth morning lessons to minimize the possibility that preparations for data assessment would disturb students' concentration on the forthcoming exam. During the fourth meeting (exactly 1 week after the exam), baseline stress reactivity was measured during a regular mathematics lesson. Finally, during the fifth meeting, conducted during a physical education lesson, students' CRF was assessed using the 20‐m shuttle run test. To ensure maximal effort from all participants, performance on the 20‐m shuttle run test contributed to their end‐of‐year grade in physical education.

### 2.3 Power Calculation

Power calculation was complicated by the fact that research in naturalistic settings is scarce. Existing studies, in which CRF was associated with HR responses to experimentally induced stress in young adults (Rimmele et al. 2007), pointed toward small‐to‐moderate effects (Cohen's *f* ^2^ = 0.12). Our power analysis (using G*Power 3.1 software; regression analysis; two‐tailed; *f* ^2^ = 0.12; *α* = 0.05; power = 0.80) indicated that a minimum of 68 participants would be required to achieve adequate statistical power. To account for an anticipated dropout rate of 30%, the targeted total sample size was set at 100 students. Assuming a class size of approximately 20 students per class and a participation rate of 60%, 8 to 9 classes were required to achieve the planned sample size.

### 2.4 Inclusion and Exclusion Criteria

To be eligible, students were required to meet the following criteria: (a) provision of signed informed consent, (b) enrollment in one of the selected classes and aged 13–16 years, (c) absence of illness at the time of the baseline data assessment and the mathematics exam, (d) participation in physical education lessons, and (e) absence of current injury. Exclusion criteria included: (a) current intake of medication with a potential influence on HR and HRV, (b) presence of any acute or chronic medical conditions that would constrain participants' physical activity. Participants who dropped out during the study or had missing HR, HRV, or CRF data, were not included in the final data analyses.

### 2.5 Measures

#### 2.5.1 Heart Rate Variability

A chest belt (Polar H10) equipped with a Polar Vantage M device (Polar Electro Europe AG, Steinhausen, Switzerland) was used to record beat‐to‐beat data (RR intervals). Off‐line processing of collected data was conducted using Kubios HRV Analysis Software 3.0.2 (The Biomedical Signal and Medical Imaging Analysis Group, Department of Applied Physics, University of Kuopio, Finland). A 4 Hz cubic spline interpolation was used to convert HRV time series resulting from RR‐intervals to equidistantly sampled series. To remove slow nonstationary trends from the signal, a linear detrend correction based on smoothness priors regularization (0.001 Hz cutoff) was applied to the R‐R series. Fast‐Fourier transformation via Welch's periodogram (300 s with 50% overlap) was used for epochs of identical length. The resulting spectrum estimates were divided into very low frequency (VLF: 0–0.04 Hz), low frequency (LF: 0.04–0.15 Hz), and high frequency (HF: 0.15–0.4 Hz) bands. Various measures of task‐related HRV were used as a dependent variable, including LF, HF, LF/HF ratio, RMSSD (root mean square of successive differences) and SDNN (standard deviation of normal‐to‐normal RR‐intervals). The following segments were generated during the stress conditions: (a) beginning of the first lesson, (b) beginning of the break before the exam, (c) beginning of the exam lesson, (d) beginning of the exam, (e) end of the exam, (f) end of the exam lesson, and (g) end of the assessment after the last morning lesson. The following segments were generated during the baseline condition: (a) beginning of the first lesson, (b) beginning of the break before the mathematics lesson, (c) beginning of the mathematic lesson, (d) end of the mathematics lesson, and (e) end of the assessment after the last morning lesson. For the purpose of this study, segments d‐e (stress condition) and c‐d (baseline condition) were compared. The segment–length for the stress and baseline conditions ranged between 40 and 45 min for all participants.

#### 2.5.2 Psychological Stress Reactivity

The Multidimensional Mood Questionnaire (MDBF) and the state version of the State–Trait–Anxiety Inventory (STAI) were applied to measure affective responses before and after both the baseline assessment and stress exposure (see Supporting Information S1 for more details including references, evidence of validity and sample items for the MDBF and STAI). The 12‐item MDBF evaluates three dimensions (4 items per scale: good‐bad mood, alertness–tiredness, calmness–restlessness). Items are anchored on a 4‐point Likert‐type scale ranging from 1 (absolutely not) to 4 (very), with higher sum scores representing better mood. Six items of the state‐version of the STAI were administered to assess current anxiety states, with items being anchored on a 4‐point Likert scale ranging from 1 (almost never) to 4 (almost always). Higher sum scores are reflective of higher state anxiety. Given that the anticipation of a maths lesson/exam can influence mood states and state anxiety, we decided not to calculate pre–post difference scores, but to generate mean scores based on the pre‐ and post‐values, separately for the baseline and stress condition.

#### 2.5.3 Cardiorespiratory Fitness

To assess CRF, the 20m shuttle–run test was administered with a starting pace of 8.5 km/h (see Supporting Information S1 for more details including references and evidence of validity of the 20m shuttle–run test). Following auditory signals, the speed was steadily increased by 0.5 km/h. The test was terminated when participants were no longer able to follow the speed of the auditory signal twice in a row. The total number of fully completed 20m laps was used as a performance indicator of CRF.

#### 2.5.4 Potential Confounders

A range of potential confounders was assessed—including age, sex, nationality, socioeconomic background, test anxiety, mathematics self‐concept, previous grade in mathematics, perceived preparedness for the mathematics exam, and perceived difficulty of the exam (see Supporting Information S1 for more details including references, evidence of validity, sample items and potential relevance of variables to be considered as covariates).

### 2.6 Statistical Analyses

A series of repeated measures analyses of covariance (rANOVAs), bivariate correlations and linear (hierarchical) regression analyses were calculated to test the main hypotheses. To examine whether a real‐life stressor leads to a physiological and psychological stress reaction compared to a nonstress baseline condition, a series of rANOVAs were carried out, including a within‐factor condition (regular mathematics lesson vs. mathematics exam). To examine whether CRF is associated with physiological and psychological outcomes, bivariate correlations between CRF and each stress marker were calculated, separately for the baseline and stress condition. Finally, to find out whether CRF predicts reactivity to the mathematics exam, a series of (hierarchical) linear regression analyses were calculated. In the first step, we controlled for potential confounders; confounders were only included if they were statistically significantly associated with CRF or if they were significantly associated with the outcome under the stress condition. In the second step, we controlled for the baseline value of the outcome. In the third step, CRF was introduced in the regression equation. Separate analyses were calculated for mean HR, mean HRV (LF, HF, LF/HF, RMSSD, SDNN), and mean state anxiety and mean current mood states. An alpha‐level of *p* < 0.05 was considered as statistically significant, and all tests were conducted with SPSS (version 29, IBM Corporation, Armonk, NY, USA). Following the general guidelines of Cohen (1988), effect sizes in rANOVAs were interpreted as follows: *η* ^2^ < 0.06 (small), 0.06 ≤ *η* ^2^ < 0.14 (medium) and *η* ^2^ ≥ 0.14 (large). In correlation and regression analysis, Pearson's *r*‐coefficients and standardized regression weights (*β*) were interpreted as follows: 0.10 to 0.29 (small), 0.30 to 0.49 (medium), and ≥ 0.50 (large).

## 3 Results

### 3.1 Sample Description

In total, 120 participants from six classes were invited to take part in the study. Hereof, 81 participants (68%) provided written informed consent. While all participants completed the questionnaire, six students did not complete assessments under the stress conditions, and additional 8 students did not take part in the baseline assessment due to illness. All of the remaining participants took part in the fitness test, resulting in a final sample of 67 students (see Table 1 for sample description).

**TABLE 1:** Sample description.

Categorical variables	*N*	%				
Sex						
Male	28	41.8				
Female	39	58.2				
Nationality						
Swiss	49	73.1				
Foreign	18	26.9				
Current medication intake						
Yes^a^	10	14.9				
No	57	85.1				

Metric variables	*M*	*SD*	Actual range	Skew	Kurt	*α*
Age (in years)	15.09	0.62	14–17	1.12	2.95	—
Height (in cm)	170.84	9.70	152–198	0.55	0.32	—
Weight (in kg)	60.59	11.27	45–102	1.46	2.65	—
Body mass index (BMI) (in kg/m^2^)	20.66	2.57	15.79–27.47	0.65	0.20	—
20m shuttle‐run test (in m)	1276.12	500.06	340–2660	0.87	0.81	—
Socioeconomic status	8.45	1.49	5–10	−0.53	−0.99	—
Mathematics self‐concept	40.48	10.65	17–59	−0.42	−0.62	0.92
Test anxiety	33.96	16.15	9–76	0.95	0.26	0.92
Anticipated difficulty of exam	6.61	1.99	2–10	−0.50	0.16	—
Feeling prepared for exam	7.42	1.70	1–10	−1.26	2.87	—
End‐of‐the‐year grade in mathematics	4.81	0.61	2.5–6.0	−0.97	2.02	—

^a^ Medications reported (including reasons) were: Ibuprofen: *n* = 3 (pain); Voltaren: *n* = 1 (pain) Tretinac: *n* = 1 (acne); Curakne: *n* = 1 (acne); Premens: *n* = 1 (premenstrual pain); Similasan: *n* = 1 (hay fever); Ferro Sanol: *n* = 1 (iron deficiency); Movicol; *n* = 1 (constipation).

Female students were slightly overrepresented in the sample (58% vs. 42%). Seventy‐three percent hold Swiss nationality, and 15% (*n* = 10) reported current medication intake. None of the students had to be excluded due to current medication intake as none of the reported medication seemed to have a clear impact on HR or HRV (see Table 1 for more information). Students had a mean age of 15.09 ± 0.62 years, and their mean body mass index (BMI) was 20.66 ± 2.57 kg/m^2^. Mean meters completed in the 20m shuttle–run test was 1276 ± 500 (corresponding to 63.81 ± 25.00 laps). The median for the 20m shuttle–run test was 1640m for boys (82 laps) and 960m for girls (48 laps).

### 3.2 Descriptive Statistics and Differences Between Conditions

Descriptive statistics for all outcome variables are shown in Table 2. With one exception (LF/HF ratio), significant differences between conditions (stress vs. baseline) were observed between all physiological and psychological outcomes, indicating that the mathematics exam triggered substantial reactions in terms of increased HR, decreased HRV, lower mood, and increased state anxiety. The effect sizes (η^2^) showed that differences were large for most variables, with the condition factor explaining between 7% and 54% of variance.

**TABLE 2:** Descriptive statistics of outcome variables, separately for stress and baseline condition.

	Stress condition			Baseline condition				
	*M*	*SD*	*α*	*M*	*SD*	*α*	*F*	*η* ^2^
Heart rate	94.18	13.55	—	86.19	11.46	—	42.37***	0.391
Heart rate variability								
LF power (ms^2^)	1302.30	968.00	—	1923.58	1188.74	—	25.90***	0.282
HF Power (ms^2^)	626.37	1038.20	—	854.96	1013.53	—	6.54*	0.090
LF/HF	3.70	1.83	—	3.52	1.72	—	0.78	0.012
RMSSD (ms)	30.98	21.77	—	40.27	23.28	—	20.06***	0.233
SDNN	42.33	18.83	—	52.54	19.63	—	29.27***	0.307
Mood: good–bad mood								
Pre	3.72	0.79	0.83	4.09	0.62	0.83	14.06***	0.176
Post	3.58	0.95	0.89	4.25	0.59	0.81	34.75***	0.345
Average Pre‐Post	3.65	0.78	—	4.17	0.53	—	31.17***	0.321
Mood: Alertness‐tiredness								
Pre	3.37	0.83	0.78	3.54	0.78	0.79	4.98*	0.070
Post	3.27	0.92	0.80	3.60	0.85	0.78	11.99***	0.154
Average Pre–Post	3.32	0.82	—	3.57	0.77	—	11.34***	0.147
Mood: Calmness‐restlessness								
Pre	3.16	0.88	0.84	3.84	0.81	0.84	34.32***	0.342
Post	3.42	0.94	0.84	3.98	0.68	0.74	28.05***	0.298
Average Pre–Post	3.29	0.82	—	3.91	0.69	—	44.84***	0.405
State anxiety								
Pre	14.18	3.57	0.86	10.73	3.15	0.81	73.56***	0.527
Post	13.16	4.23	0.89	10.00	2.48	0.64	41.33***	0.385
Average Pre–Post	13.67	3.57	—	10.37	2.49	—	76.54***	0.537

Abbreviations: *α*, cronbach's alpha; HF, high frequency; LF, low frequency; RMSSD, root mean square of successive differences; SDNN, standard deviation of normal‐to‐normal RR‐intervals.

^*^ *p* < 0.05.

^***^ *p* < 0.001.

### 3.3 Association Between Cardiorespiratory Fitness and Potential Confounders

With one exception, no statistically significant associations were found between potential confounders and participants' CRF (*p* > 0.05; see Supporting Information S1: Tables S4.1–S4.2). The exception was that participants with higher CRF reported lower test anxiety (*r* = −0.24, *p* = 0.048).

### 3.4 Association Between Potential Confounders and Stress Markers

Associations between potential confounders and physiological and psychological stress markers are displayed in the Supporting Information S1: Tables S5.1–S5.10). These results indicate that participants with higher maths self‐concept report lower heart rate, higher SDNN, better mood, and lower state anxiety during the stress condition. While higher test anxiety scores were associated with poorer mood and higher state anxiety across the baseline and stress condition, students who perceived the exam as difficult reported poorer mood and higher state anxiety during the stress condition. Moreover, students with better end‐of‐the‐year results in mathematics reported higher scores in one (of three) mood indicator(s), but only during the regular mathematics lesson (baseline). Finally, significant sex differences were observed for two HRV markers during the baseline condition. Thus, male students had higher LF power and SDNN. Nevertheless, these differences were not observed under real‐life stress. Male students also reported better mood and lower state anxiety, with the differences in anxiety being more pronounced under stress conditions.

### 3.5 Bivariate Association Between Cardiorespiratory Fitness and Stress Markers

**FIGURE 2:**
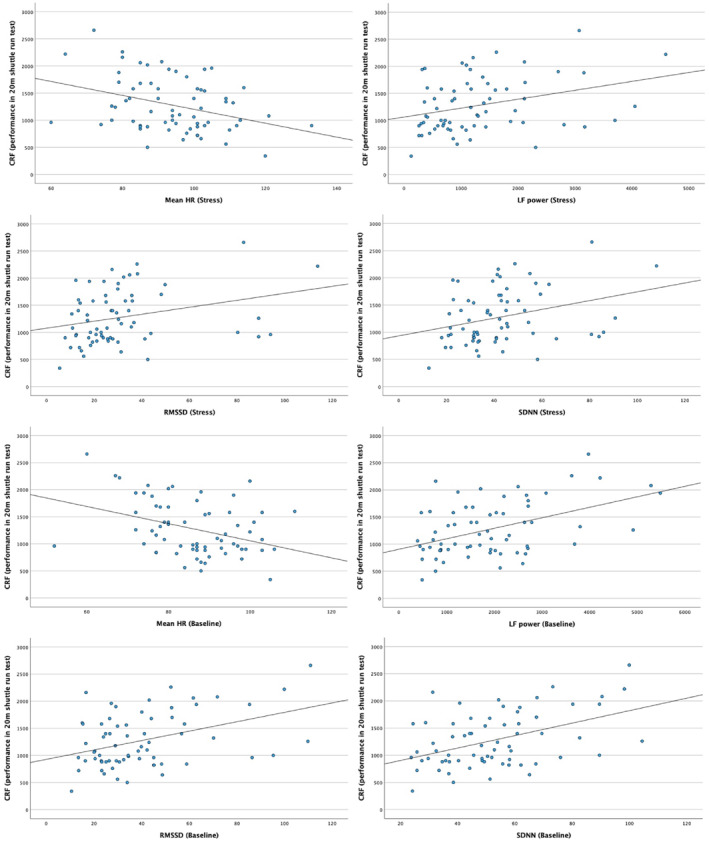
Graphical representation of the (significant) bivariate correlations between CRF and HR/HRV markers across stress and baseline conditions. CRF, cardiorespiratory fitness; HF, high frequency; LF, low frequency; RMSSD, root mean square of successive differences; SDNN, standard deviation of normal‐to‐normal RR‐intervals.

**FIGURE 3:**
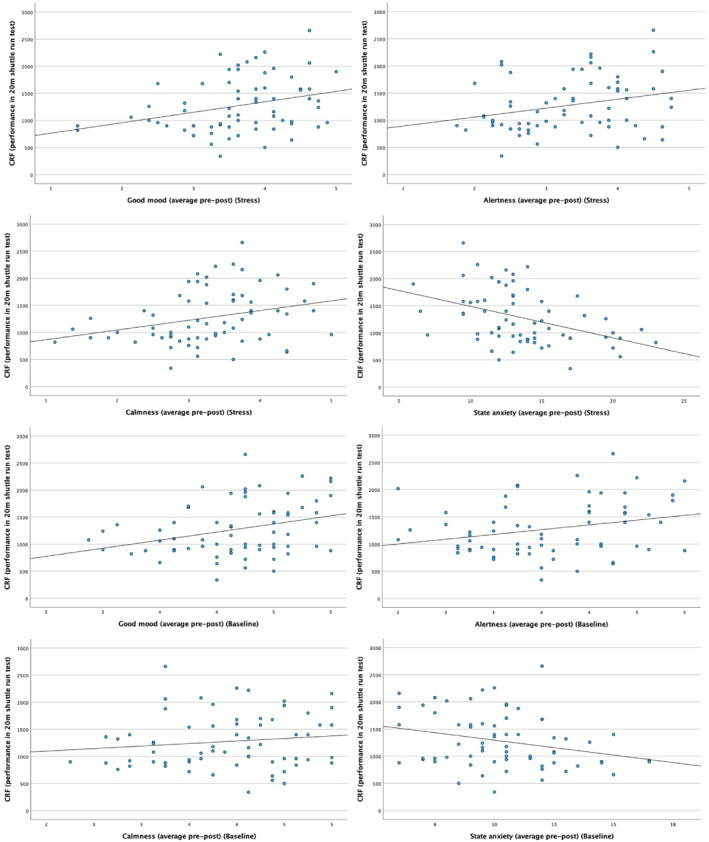
Graphical representation of the bivariate correlations between CRF and markers of mood and state anxiety across stress and baseline conditions. CRF, Cardiorespiratory fitness.

Table 3 shows the bivariate correlations between participants' CRF and physiological and psychological outcomes, separately for baseline and stress condition. These results highlight that adolescents with better CRF had lower heart rate, higher LF power, higher HF power (during baseline condition only), higher RMSSD and higher SDNN (Figure 2). Moreover, CRF was positively correlated with almost all markers of current mood states (across baseline and stress condition), whereas a negative correlation emerged between CRF and state anxiety (Figure 3). The significant correlations were in the small‐to‐medium range.

**TABLE 3:** Bivariate correlations (Pearson's *r*) between cardiorespiratory fitness and stress markers, separately for stress and baseline condition.

	Cardiorespiratory fitness (CRF)	
	Baseline condition	Stress condition
Heart rate	−0.36**	−0.35**
Heart rate variability		
LF power (ms^2^)	0.46***	0.32**
HF Power (ms^2^)	0.38***	0.21
LF/HF	−0.11	−0.05
RMSSD (ms)	0.40***	0.28*
SDNN	0.45***	0.31*
Mood		
Good–bad (average pre‐post)	0.31**	0.30*
Alertness–tiredness (average pre‐post)	0.27*	0.27*
Calmness–restlessness (average pre‐post)	0.13	0.29*
State anxiety (average pre‐post)	−0.27*	−0.42***

Abbreviations: HF, high frequency; LF, low frequency; RMSSD, root mean square of successive differences; SDNN, standard deviation of normal‐to‐normal RR‐intervals.

^*^ *p* < 0.05.

^**^ *p* < 0.01.

^***^ *p* < 0.001.

### 3.6 Predication of Stress Reactivity by Cardiorespiratory Fitness

Table 4 shows the results of the linear hierarchical regression analysis. After controlling for relevant confounders and baseline scores (nonstress control condition), CRF did not predict any of the physiological and psychological markers of stress reactivity. The total models explained between 35% and 59% of variance. The overall pattern indicated that baseline values showed the strongest predictive power, with significant regression weights (*β*) ranging from 0.34 to 0.78 (medium‐to‐strong association). This indicates that students who presented with higher HR, HRV, mood, or state anxiety levels during the regular mathematics lesson (baseline) also had higher scores for these measures during the exam situation. Beyond these influences, CRF did not account for additional explained variance in stress reactivity.

**TABLE 4:** Linear hierarchical regression analyses predicting physiological and psychological markers during the stress condition with cardiorespiratory fitness (step 3), after controlling for potential confounders (step 1), and baseline values assessed during the nonstress control condition (step 2).

	HR		LF power		HF power		LF/HF		RMSSD		SDNN	
	*β*	Δ*R* ^2^	*β*	Δ*R* ^2^	*β*	Δ*R* ^2^	*β*	Δ*R* ^2^	*β*	Δ*R* ^2^	*β*	Δ*R* ^2^
Step 1 (confounders)		0.064		—		—		—		—		0.058
Sex	—		—		—		—		—		—	
Maths self‐concept	−0.14		—		—		—		—		0.16	
Step 2		0.434***		0.345***		0.556***		0.336***		0.515***		0.428***
Baseline score of outcomes	0.64***		0.56***		0.78***		0.58***		0.72***		0.67***	
Step 3		0.007		0.003		0.007		0.000		0.000		0.001
Cardiorespiratory fitness	−0.10		0.06		−0.09		0.01		−0.01		−0.03	
Total model	*F* = 21.42***, *R* ^2^ = 0.505		*F* = 17.07***, *R* ^2^ = 0.348		*F* = 41.23***, *R* ^2^ = 0.563		*F* = 16.22***, *R* ^2^ = 0.336		*F* = 34.01***, *R* ^2^ = 0.515		*F* = 19.85***, *R* ^2^ = 0.486	

	**Good‐bad mood** ^a^		**Alertness‐tiredness** ^a^		**Calmness‐restlessness** ^a^		**State anxiety** ^a^	
	***β***	**Δ*R*** ^**2**^	***β***	**Δ*R*** ^**2**^	***β***	**Δ*R*** ^**2**^	***β***	**Δ*R*** ^**2**^
Step 1 (confounders)		0.385***		0.315***		0.328***		0.486***
Sex	−0.10		−0.06		−0.06		0.20	
Maths self‐concept	0.02		—		−0.04		−0.03	
Test anxiety	−0.34		−0.08		−0.21		0.29	
Anticipated difficulty of exam	−0.30		−0.23		−0.32		0.29	
Step 2		0.027		0.268***		0.117***		0.106***
Baseline score of outcomes	0.18		0.63***		0.40***		0.34***	
Step 3		0.007		0.002		0.017		0.010
Cardiorespiratory fitness	0.11		0.05		0.17		−0.13	
Total model	*F* = 7.20***, *R* ^2^ = 0.418		*F* = 17.18***, *R* ^2^ = 0.585		*F* = 8.60***, *R* ^2^ = 0.462		*F* = 15.15***, *R* ^2^ = 0.563	

*Note:* Age, body mass index, socioeconomic background, feeling prepared for the exam, end‐of‐the‐year grade in mathematics, and nationality were not considered as potential confounders as these variables were neither associated with cardiorespiratory fitness nor with any of the physiological and psychological outcomes during the stress condition.

Abbreviations: HF, high frequency; HR, heart rate; LF, low frequency; RMSSD, root mean square of successive differences; SDNN, standard deviation of normal‐to‐normal RR‐intervals.

^a^ The average of the pre and post scores during the stress and baseline condition were used as predictors (step 2) and outcomes.

^***^ *p* < 0.001.

## 4 Discussion

The key finding of this study is that a mathematics exam, as a personally meaningful real‐life stressor, elicits pronounced physiological and psychological stress responses among adolescent secondary school students. Better CRF was associated with lower HR, higher HRV, better mood and lower state anxiety across the baseline and stress condition. After controlling for relevant confounders and baseline scores of the outcomes, CRF did not explain additional variance in the reactivity of the physiological and psychological markers.

Our results support the notion that academic exams induce substantial stress reactions, supporting school‐related performance demands as a source of distress. While activation of the sympathoadrenal system is functionally adaptive, facilitating cellular and systemic function (Chrousos 2009), academic stressors may also contribute to increased allostatic load (McEwen 2013), which could entail long‐term negative consequences for those affected (Juster et al. 2010). HRV is considered a viable indicator of the activity of the parasympathetic and sympathetic nervous system (Shaffer and Ginsberg 2017). Specifically, the sympathetic nervous system is responsible for the initiation of a fight‐or‐flight reaction when people are exposed to stress, which results in an increase in HR and a decrease in HRV (Salmio et al. 2024). Although higher HRV is reflective of adequate autonomic control, positive adaptions of the organism and sufficient energy reserves, lower HRV can reflect increased sympathetic activation, disturbed autonomous nervous system (ANS) regulation, inadequate adaptation of the cardiovascular system, chronic stress, and depleted energy reservoirs (Shaffer and Ginsberg 2017).

Given that chronically reduced HRV is detrimental to long‐term health (Arakaki et al. 2023), and that stress has a far‐reaching impact on secondary school students' health, wellbeing, and future success (Pascoe et al. 2020), it is crucial to identify strategies to support students in coping with academic stress. Two possible avenues to achieve this goal include the instruction of coping strategies (Lang et al. 2016) or biofeedback training (Goessl et al. 2017). An alternative strategy might be to promote students' CRF.

The findings of our study further reinforce that better CRF is associated with more favorable HRV profiles (i.e., higher parasympathetic activity) in adolescents (Oliveira et al. 2017). This can be attributed to physiological adaptations resulting from regular engagement in physical activity (including endurance, resistance, high‐intensity, and coordinative exercises), which enhance HRV parameters (Grassler et al. 2021). Exercise‐based adaptation in HRV can be ascribed to different physiological mechanisms (Grassler et al. 2021), including a reduction in sympathetic influence on HR via decreased plasma noradrenaline concentration (Kiviniemi et al. 2010), suppression of angiotensin II, which in turn might increase the parasympathetic tone on the HR (Routledge et al. 2010), or improved baroreflex functional capacity as a result of nitric oxide synthesis and induction of greater carotid artery distensibility (Bhati et al. 2019). In light of these mechanisms, the promotion of regular exercise training is an important endeavor, as better CRF during youth is associated with a reduced risk of physical health complications later in life (Garcia‐Hermoso et al. 2020).

Although our results align with previous investigations showing that people with better fitness levels tend to be less anxious (Hallgren et al. 2020), our expectation that better CRF would predict a more favorable reaction to a real‐life stressor (after controlling for relevant confounders and baseline scores) was not supported. This is consistent with some previous studies in children and young people, where CRF did not moderate the autonomous stress reactivity in laboratory settings (Childs and de Wit 2014; Gerber, Ludyga, et al. 2017b). Using the Trier Social Stress Test (TSST), Mücke et al. (2021) found that CRF did not account for variance in psychological stress reactivity, whereas better CRF was associated with lower ANS reactivity, as indicated by alpha‐amylase concentration. With regard to the influence of CRF on stress reactivity during exposure to real‐life stressors, von Haaren et al. (2016) found that a 20‐week exercise–training program had a positive effect on HRV in university students and could cushion physiological stress reactivity during semester exams. Nevertheless, it is important to highlight that the absence of statistically significant regression weights in our regression analyses does not indicate that CRF does not mitigate the harmful effects of stress. Rather, as shown in our study, CRF was associated with lower HR, higher HRV, better mood and lower state anxiety independent of students' stress levels. Thus, from a diathesis–stress model perspective, better CRF might reduce the risk that a certain vulnerability threshold is exceeded, beyond which stress begins to be harmful (Nielsen et al. 2020). At present, however, it is difficult to determine where the negative effects of reduced HRV start to manifest. Although attempts were made to establish norms of decreased HRV (Shaffer and Ginsberg 2017), it is uncertain whether these cut‐points are applicable to secondary school students.

### 4.1 Strengths and Limitations

To our knowledge, our study is among the first to test the association between CRF and the stress response to a personally meaningful, real‐life stressor among adolescents. Our main analyses were controlled for relevant confounders and baseline scores, and we used both physiological and psychological indicators of stress reactivity. In order to rule out that associations are driven by pharmacological factors, current intake of medication with a potential effect on HR/HRV was used as an exclusion criterion. While it is necessary to compare outcomes with a baseline condition when examining stress reactivity, the fact that we used a regular mathematics lesson as a baseline comparator needs to be considered as regular lessons can already elicit low‐level stress and induce low‐level stress reactivity. In turn, this elevated baseline level can mask interaction effects. Given the voluntary nature of participation, we can also not fully rule out that students with higher test anxiety or poorer math skills were less inclined to participate. We also acknowledge that we did not employ a standardized math test and that both the content and likely also the difficulty of the exams may have varied. Although the 20m shuttle–run test is well‐established in child and adolescent research (see Supporting Information S1), differences in motivation and other potential sources of measurement error may have influenced performance and thus, our findings. Finally, fitness levels were relatively high in the present study compared to international samples (Tomkinson et al. 2017), and based on their absolute HRV scores, our sample appeared to be in good health condition (Shaffer and Ginsberg 2017). Consequently, a ceiling effect cannot be ruled out. The hypothesized association between CRF and stress reactivity may be more readily demonstrated in a sample exhibiting greater variation in fitness levels and/or health status. Finally, we also acknowledge that we did not control for the menstrual cycle of the female participants.

## 5 Conclusion

In this study, we studied whether CRF predicts HR, HRV, current mood states, and state anxiety during a real‐life academic stressor (maths exam) after controlling for relevant confounders and baseline values assessed during a regular mathematics lesson. As shown in our study, exposure to a mathematics exam elicited substantial stress responses in adolescent secondary school students. While better CRF was associated with favorable physiological and psychological states, this relationship appeared to be independent of students' current stress exposure. The identification of factors that contribute to an adaptive response to acute stressors is of considerable importance, as prolonged dysregulation of the stress response has been linked to adverse health outcomes, and academic stress has been identified as a risk factor for subsequent mental health problems in adolescents. Further research employing other ecologically valid stressors is warranted to better understand the relationship between CRF and physiological and psychological stress reactivity in real life.

## Funding

The authors have nothing to report.

## Ethics Statement

The procedures of this study were approved by the responsible ethical review board prior to data collection (EKNZ, 2022‐01438).

## Consent

All students and their parents/legal guardians provided written informed consent.

## Conflicts of Interest

The authors declare no conflicts of interest.

## Permission to Reproduce Material From Other Sources

The authors have nothing to report.

## Supporting information


Supporting Information S1

## Data Availability

The data that support the findings of this study are available on request from the corresponding author. The data are not publicly available due to privacy or ethical restrictions.
