# Supplementary material for: Does Cardiorespiratory Fitness Predict the Physiological and Psychological Stress Response to a Mathematics Exam in Secondary High School Students?
Source: Eur J Sport Sci. 2026 Apr 8;26(5):e70172. doi: 10.1002/ejsc.70172 (PMC13060642; doi:10.1002/ejsc.70172)
Supplement: Supplementary file 1 — Supporting Information S1 [file EJSC-26-e70172-s001.docx]

**Supplement**

**Does cardiorespiratory fitness predict the physiological and psychological stress response to a mathematics exam in secondary high school students?**

**1. Description of potential confounders**

**Socioeconomic background** was assessed with the 4-item Family Affluence Scale (Torsheim, Cavallo, Levin, Schnohr, Mazur, Niclasen, & Currie, 2016), which is a brief self-report measure developed within the Health Behaviour in School-aged Children **(HBSC)** study to assess adolescents’ material family wealth. Items included focus on car ownership, own bedroom, computer ownership and holidays abroad. Items can take values between 1 and 3, with higher sum (possible range: 4-12) scores representing higher socio-economic status. Evidence of the validity of this instrument has been presented previously (Torsheim, Cavallo, Levin, Schnohr, Mazur, Niclasen, Currie, et al., 2016). Socioeconomic background was considered as a covariate because this family affluence proved to be associated with stress, mental health and risk behavior in adolescents (Elgar et al., 2013; Haugan et al., 2021; Schelleman-Offermans et al., 2022).

**Maths self-concept** was assessed with 10 items from the Self-Description Questionnaire II(Marsh, 1990a, 1990b). This scale measures students’ self-perception about their mathematics skills. All items were anchored on a 6-point Likert-scale ranging from 1 (not at all true) to 6 (completely true). Sample items are “I am good at mathematics” or “Mathematics is one of my best subjects”. Sum scores were built (possible range: 10-60), with higher scores reflecting a more favorable self-perception. Evidence for the validity of the SDQ II has been reported in previous studies(Gilman et al., 1999; Guerin et al., 2003; Marsh et al., 2005). Maths self-concept was considered as a covariate because this variable proved to be associated with maths anxiety, general test anxiety, self-efficacy and academic achievement (Ahmed et al., 2012; Ding et al., 2024; Timmerman et al., 2017).

**Test anxiety** was assessed with the Children’s Test Anxiety Scale (CTAS)(Wren & Benson, 2004). This instrument measures students’ anxiety to engage in academic tests. The CTAS consists of 30 items and assess components such as worrying thoughts (13 items), autonomic reaction (9 items) and off-task behavior (8 items). Sample items are “I think I am going to get a bad grade”, “My head hurts” or “I look around the room”. Items are answered on a 4-point Likert-type scales ranging from 0 (almost never) to 3 (almost always). In the present study, the 30 items were summed to obtain a total score (0-90), with higher values reflecting higher test anxiety. Evidence for the validity and reliability of this instrument has been presented in prior research(Adul Kader & Eissa, 2014; Shoahosseini & Baghaei, 2020; Wren & Benson, 2004). Test anxiety was considered as a covariate because this variable proved to be associated with increase academic stress and mental health problems among student populations (Putwain et al., 2021; Zheng et al., 2023).

**Anticipated difficulty** and **feeling prepared for the exam** were assessed with two 1-item questions ranging from 1 (not at all difficult, not at all prepared) to 10 (very difficult, very well prepared). **End-of-the-year grades** for the previous grade were self-reported by the students. In Switzerland, end-of-the-year grades can vary between 1 and 6. Students need a 4 to pass, a 5 is good and a 6 means excellent (pass with distinction). These variables were considered as skill level and study preparation proved to have an influence on test anxiety (Torrano et al., 2020; Yusefzadeh et al., 2019).

**Sex** was considered as covariate because girls generally report more school stress than boys (Garcia-Moya et al., 2025; Högberg, 2021; Huan et al., 2008). Similar **age** was considered as a relevant covariate as school stress seems to increase with age (Badura et al., 2024; Klinger et al., 2015). Research also shows that **overweight students** (Kaczmarek & Trambacz-Oleszak, 2021; Kanellopoulou et al., 2022) and **students with migration background** (Alivernini et al., 2020; Vogelaar et al., 2024) tend to perceive more school stress. Accordingly, BMI and nationality were considered as potential confounders, as well.

**2. Description of markers to operationalize psychological stress reactivity**

**Current mood states** before and after the baseline assessment and stress exposure were assessed with the Multidimensional Mood Questionnaire (MDBF) (Steyer et al., 1997)and the state version of the State-Trait-Anxiety Inventory (STAI) (Schwenkmezger et al., 1992; Spielberger et al., 1970). The MDBF inquires 12 different mood states, representing three dimensions (four items per scale: good-bad mood, alertness-tiredness, calmness-restlessness). All items are based on the same stem: “What is your mood at the moment”. Examples of assessed items are: satisfied, well-rested, calm. Each item is answered on a 4-point Likert-type scale from 1 (absolutely not) to 4 (very). Scale scores are built by summing the scores of the four respective items. The MDBF proved to have adequate psychometric properties in previous studies (Hinz et al., 2012; Steyer et al., 1997).

**State anxiety** was assessed with the state-version of the STAI (s-STAI). The original instrument is composed of 20 items that assess anxiety states. However, since there is some overlap between the items of the MDBF and the STAI, and since some of the STAI items are difficult to understand for children/adolescents, we decided to use a simple 6-item of the s-STAI to measure current anxiety states (Nilsson et al., 2012). The following items were used: (1) “I feel calm”, (2) “I am tense”, (3) “I feel upset”, (4) “I feel self-confident”, (5) “I feel content”, (6) “I am worried”. All items were answered on a 4-point Likert scale from 1 (almost never) to 4 (almost always). The items were summed to calculate an overall score, with higher scores reflecting higher state anxiety. Evidence that the STAI has acceptable psychometric properties has been published previously (Laux et al., 1981; Schwenkmezger et al., 1992; Thomas & Cassady, 2021).

**3. Assessment of cardiorespiratory fitness**

Cardiorespiratory fitness was assessed with the 20m shuttle run test (Leger et al., 1988), starting with a pace of 8.5 km/h. The speed was steadily increased by 0.5 km/h, following sound signals. The test will be ﬁnished when children are no longer able to follow the speed of the sound signal twice in a row. The total number of fully completed 20 m laps was noted as a performance indicator of CRF. The 20 m Shuttle run is a reliable and broadly validated ﬁeld test to assess cardiorespiratory fitness among children and adolescents(Lang, 2018; Lang et al., 2017; Lang, Tomkinson, et al., 2018; Lang, Wolfe Philips, et al., 2018). The test is inexpensive, time-efficient, and feasible for large-scale school or epidemiological settings. It requires minimal equipment, can be administered to groups simultaneously, and demonstrates moderate-to-high criterion validity against directly measured VO₂peak/VO₂max at the group level (Mayorga-Vega et al., 2015). Test–retest reliability is generally acceptable when standardized procedures are followed (Leger et al., 1988). Accordingly, major scientific bodies recognize the 20m shuttle run test as a practical field indicator of CRF in youth (Ortega et al., 2025; Ross et al., 2016). However, important limitations must be considered. Performance reflects not only aerobic capacity but also motivation, pacing ability, change-of-direction skill, and anaerobic contribution, which may introduce measurement error, particularly in less mature or less coordinated adolescents. Furthermore, prediction equations for estimating VO₂max from shuttle performance show considerable variability and individual-level error, limiting clinical interpretation (Mayorga-Vega et al., 2015; Ruiz et al., 2009). Body mass may further confound results, as weight-bearing performance can disadvantage adolescents with overweight or obesity (Armstrong & Welsman, 2019). Consequently, experts recommend reporting completed stages or shuttles rather than relying on derived VO₂values and emphasize cautious interpretation at the individual level (Ross et al., 2016). Overall, the 20m shuttle run test is a valid and practical group-level field measure of CRF in adolescents, but its physiological specificity and predictive precision are limited.

**4. Association between cardiorespiratory fitness and potential confounders**

**Table S4.1.** Bivariate correlations between CRF and metric confounders

|  | Cardiorespiratory fitness (CRF) |
| --- | --- |
| Age | *r* = -0.03, *p* = 0.803 |
| Body mass index (BMI) | *r* = -0.19, *p* = 0.120 |
| Socioeconomic status | *r* = 0.05, *p* = 0.686 |
| Maths self-concept | *r* = 0.186, *p* = 0.131 |
| Test anxiety | ***r* = -0.24, *p* = 0.048** |
| Anticipated difficulty of exam | *r* = 0.05, *p* = 0.716 |
| Feeling prepared for exam | *r* = 0.06, *p* = 0.652 |
| End-of-the-year grade in maths | *r* = 0.06, *p* = 0.634 |

**Table S4.2.** Differences in CRF for categorical confounders

|  | Cardiorespiratory fitness (CRF) |  |
| --- | --- | --- |
| Sex |  |  |
| Male | *M* = 1647.86, *SD* = 476.60 | ***F* = 43.83, *p* < 0.001, η^2^ = 0.403** |
| Female | *M* = 1009.23, *SD* = 313.04 |  |
| Nationality |  |  |
| Swiss | *M* = 1247.78, *SD* = 454.11 | *F* = 0.08, *p* = 0.781, η^2^ = 0.001 |
| Foreign | *M* = 1286.53, *SD* = 519.90 |  |

**5. Association between potential confounders and physiological and psychological outcomes**

**Table S5.1.** Bivariate correlations between age and physiological and psychological outcomes

|  | Age | |
| --- | --- | --- |
|  | Baseline condition | Stress condition |
| Heart rate | *r* = -0.06, *p* = 0.646 | *r* = 0.10, *p* = 0.434 |
| Heart rate variability |  |  |
| LF power (ms^2^) | *r* = -0.09, *p* = 0.493 | *r* = -0.15, *p* = 0.212 |
| HF power (ms^2^) | *r* = -0.02, *p* = 0.888 | *r* = -0.14, *p* = 0.252 |
| LF/HF | *r* = 0.00, *p* = 0.991 | *r* = -0.04, *p* = 0.736 |
| RMSSD (ms) | *r* = -0.03, *p* = 0.822 | *r* = -0.14, *p* = 0.244 |
| SDNN | *r* = -0.07, *p* = 0.576 | *r* = -0.15, *p* = 0.221 |
| Mood: Average Pre-Post |  |  |
| Good-bad | *r* = 0.13, *p* = 0.310 | *r* = 0.13, *p* = 0.304 |
| Alertness-tiredness | *r* = 0.17, *p* = 0.172 | *r* = 0.23, *p* = 0.066 |
| Calmness-restlessness | *r* = 0.10, *p* = 0.431 | *r* = 0.10, *p* = 0.434 |
| Anxiety | *r* = -0.16, *p* = 0.199 | *r* = -0.18, *p* = 0.142 |

*Notes:* LF=Low frequency, HF=High frequency, RMSSD=Root mean square of successive differences, SDNN=Standard deviation of normal-to-normal RR-intervals.

**Table S5.2.** Bivariate correlations between body mass index (BMI) and physiological and psychological outcomes

|  | BMI | |
| --- | --- | --- |
|  | Baseline condition | Stress condition |
| Heart rate | *r* = -0.08, *p* = 0.510 | *r* = -0.07, *p* = 0.558 |
| Heart rate variability |  |  |
| LF power (ms^2^) | *r* = 0.00, *p* = 0.995 | *r* = -0.02, *p* = 0.859 |
| HF power (ms^2^) | *r* = -0.03, *p* = 0.792 | *r* = 0.05, *p* = 0.705 |
| LF/HF | *r* = 0.09, *p* = 0.492 | *r* = 0.09, *p* = 0.461 |
| RMSSD (ms) | *r* = -0.03, *p* = 0.839 | *r* = -0.01, *p* = 0.946 |
| SDNN | *r* = 0.02, *p* = 0.875 | *r* = -0.02, *p* = 0.897 |
| Mood: Average Pre-Post |  |  |
| Good-bad | *r* = 0.05, *p* = 0.673 | *r* = 0.10, *p* = 0.426 |
| Alertness-tiredness | *r* = 0.04, *p* = 0.772 | *r* = 0.02, *p* = 0.896 |
| Calmness-restlessness | *r* = 0.09, *p* = 0.479 | *r* = 0.08, *p* = 0.507 |
| Anxiety | *r* = -0.09, *p* = 0.471 | *r* = -0.10, *p* = 0.404 |

*Notes:* LF=Low frequency, HF=High frequency, RMSSD=Root mean square of successive differences, SDNN=Standard deviation of normal-to-normal RR-intervals.

**Table S5.3.** Bivariate correlations between socioeconomic background and physiological and psychological outcomes

|  | Socioeconomic background | |
| --- | --- | --- |
|  | Baseline condition | Stress condition |
| Heart rate | *r* = 0.14, *p* = 0.276 | *r* = 0.15, *p* = 0.238 |
| Heart rate variability |  |  |
| LF power (ms^2^) | *r* = 0.04, *p* = 0.756 | *r* = 0.08, *p* = 0.547 |
| HF power (ms^2^) | *r* = -0.08, *p* = 0.510 | *r* = -0.06, *p* = 0.620 |
| LF/HF | *r* = 0.08, *p* = 0.545 | *r* = -0.08, *p* = 0.508 |
| RMSSD (ms) | *r* = -0.09, *p* = 0.476 | *r* = -0.05, *p* = 0.663 |
| SDNN | *r* = -0.01, *p* = 0.934 | *r* = 0.03, *p* = 0.826 |
| Mood: Average Pre-Post |  |  |
| Good-bad | *r* = 0.16, *p* = 0.194 | *r* = 0.13, *p* = 0.309 |
| Alertness-tiredness | *r* = 0.12, *p* = 0.336 | *r* = 0.17, *p* = 0.164 |
| Calmness-restlessness | *r* = 0.10, *p* = 0.431 | *r* = -0.06, *p* = 0.641 |
| Anxiety | *r* = -0.12, *p* = 0.348 | *r* = 0.01, *p* = 0.948 |

*Notes:* LF=Low frequency, HF=High frequency, RMSSD=Root mean square of successive differences, SDNN=Standard deviation of normal-to-normal RR-intervals.

**Table S5.4.** Bivariate correlations between mathematics self-concept and physiological and psychological outcomes

|  | Mathematics self-concept | |
| --- | --- | --- |
|  | Baseline condition | Stress condition |
| Heart rate | *r* = -0.15, *p* = 0.227 | ***r* = -0.25, *p* = 0.039** |
| Heart rate variability |  |  |
| LF power (ms^2^) | *r* = 0.09, *p* = 0.496 | *r* = 0.24, *p* = 0.054 |
| HF power (ms^2^) | *r* = 0.12, *p* = 0.352 | *r* = 0.21, *p* = 0.094 |
| LF/HF | *r* = -0.07, *p* = 0.550 | *r* = 0.00, *p* = 0.984 |
| RMSSD (ms) | *r* = 0.15, *p* = 0.241 | *r* = 0.22, *p* = 0.072 |
| SDNN | *r* = 0.12, *p* = 0.320 | ***r* = 0.24, *p* = 0.050** |
| Mood: Average Pre-Post |  |  |
| Good-bad | ***r* = 0.25, *p* = 0.044** | ***r* = 0.34, *p* = 0.005** |
| Alertness-tiredness | *r* = 0.14, *p* = 0.271 | *r* = 0.23, *p* = 0.056 |
| Calmness-restlessness | *r* = 0.15, *p* = 0.218 | ***r* = 0.26, *p* = 0.036** |
| Anxiety | *r* = -0.19, *p* = 0.125 | ***r* = -0.38, *p* = 0.002** |

*Notes:* LF=Low frequency, HF=High frequency, RMSSD=Root mean square of successive differences, SDNN=Standard deviation of normal-to-normal RR-intervals.

**Table S5.5.** Bivariate correlations between test anxiety and physiological and psychological outcomes

|  | Test anxiety | |
| --- | --- | --- |
|  | Baseline condition | Stress condition |
| Heart rate | *r* = 0.02, *p* = 0.891 | *r* = -0.15, *p* = 0.232 |
| Heart rate variability |  |  |
| LF power (ms^2^) | *r* = -0.02, *p* = 0.904 | *r* = -0.07, *p* = 0.573 |
| HF power (ms^2^) | *r* = -0.03, *p* = 0.792 | *r* = -0.03, *p* = 0.799 |
| LF/HF | *r* = 0.02, *p* = 0.863 | *r* = 0.02, *p* = 0.859 |
| RMSSD (ms) | *r* = -0.06, *p* = 0.651 | *r* = -0.06, *p* = 0.626 |
| SDNN | *r* = -0.03, *p* = 0.796 | *r* = -0.08, *p* = 0.531 |
| Mood: Average Pre-Post |  |  |
| Good-bad | ***r* = -0.34, *p* = 0.005** | ***r* = -0.51, *p* < 0.001** |
| Alertness-tiredness | ***r* = -0.36, *p* = 0.003** | ***r* = -0.36, *p* = 0.002** |
| Calmness-restlessness | ***r* = -0.38, *p* = 0.002** | ***r* = -0.44, *p* < 0.001** |
| Anxiety | ***r* = 0.39, *p* = 0.001** | ***r* = 0.57, *p* < 0.001** |

*Notes:* LF=Low frequency, HF=High frequency, RMSSD=Root mean square of successive differences, SDNN=Standard deviation of normal-to-normal RR-intervals.

**Table S5.6.** Bivariate correlations between anticipated difficulty of exam and physiological and psychological outcomes

|  | Perceived difficulty of exam | |
| --- | --- | --- |
|  | Baseline condition | Stress condition |
| Heart rate | *r* = 0.22, *p* = 0.080 | *r* = 0.20, *p* = 0.104 |
| Heart rate variability |  |  |
| LF power (ms^2^) | *r* = -0.01, *p* = 0.921 | *r* = -0.05, *p* = 0.701 |
| HF power (ms^2^) | *r* = -0.05, *p* = 0.685 | *r* = -0.11, *p* = 0.397 |
| LF/HF | *r* = 0.09, *p* = 0.453 | *r* = -0.04, *p* = 0.780 |
| RMSSD (ms) | *r* = -0.11, *p* = 0.381 | *r* = -0.14, *p* = 0.269 |
| SDNN | *r* = -0.06, *p* = 0.632 | *r* = -0.11, *p* = 0.397 |
| Mood: Average Pre-Post |  |  |
| Good-bad | *r* = 0.04, *p* = 0.781 | ***r* = -0.34, *p* = 0.005** |
| Alertness-tiredness | *r* = -0.06, *p* = 0.615 | ***r* = -0.28, *p* = 0.023** |
| Calmness-restlessness | *r* = 0.02, *p* = 0.892 | ***r* = -0.32, *p* = 0.008** |
| Anxiety | *r* = -0.06, *p* = 0.649 | ***r* = 0.32, *p* = 0.008** |

*Notes:* LF=Low frequency, HF=High frequency, RMSSD=Root mean square of successive differences, SDNN=Standard deviation of normal-to-normal RR-intervals.

**Table S5.7.** Bivariate correlations between feeling of being prepared for exam and physiological and psychological outcomes

|  | Feeling of being prepared for exam | |
| --- | --- | --- |
|  | Baseline condition | Stress condition |
| Heart rate | *r* = -0.11, *p* = 0.399 | *r* = -0.10, *p* = 0.402 |
| Heart rate variability |  |  |
| LF power (ms^2^) | *r* = -0.10, *p* = 0.428 | *r* = -0.08, *p* = 0.524 |
| HF power (ms^2^) | *r* = -0.02, *p* = 0.878 | *r* = -0.02, *p* = 0.898 |
| LF/HF | *r* = -0.14, *p* = 0.243 | *r* = -0.03, *p* = 0.782 |
| RMSSD (ms) | *r* = 0.02, *p* = 0.876 | *r* = 0.02, *p* = 0.861 |
| SDNN | *r* = -0.04, *p* = 0.780 | *r* = -0.02, *p* = 0.863 |
| Mood: Average Pre-Post |  |  |
| Good-bad | *r* = -0.24, *p* = 0.055 | *r* = 0.05, *p* = 0.704 |
| Alertness-tiredness | *r* = -0.07, *p* = 0.597 | *r* = 0.10, *p* = 0.450 |
| Calmness-restlessness | *r* = -0.11, *p* = 0.394 | *r* = 0.06, *p* = 0.660 |
| Anxiety | *r* = 0.14, *p* = 0.274 | *r* = -0.15, *p* = 0.219 |

*Notes:* LF=Low frequency, HF=High frequency, RMSSD=Root mean square of successive differences, SDNN=Standard deviation of normal-to-normal RR-intervals.

**Table S5.8.** Bivariate correlations between end-of-the-year grade in mathematics and physiological and psychological outcomes

|  | End-of-the-year grade in mathematics | |
| --- | --- | --- |
|  | Baseline condition | Stress condition |
| Heart rate | *r* = -0.09, *p* = 0.490 | *r* = -0.14, *p* = 0.262 |
| Heart rate variability |  |  |
| LF power (ms^2^) | *r* = -0.08, *p* = 0.544 | *r* = 0.03, *p* = 0.834 |
| HF power (ms^2^) | *r* = -0.07, *p* = 0.571 | *r* = 0.07, *p* = 0.590 |
| LF/HF | *r* = 0.03, *p* = 0.793 | *r* = 0.02, *p* = 0.880 |
| RMSSD (ms) | *r* = -0.04, *p* = 0.734 | *r* = 0.07, *p* = 0.593 |
| SDNN | *r* = -0.07, *p* = 0.598 | *r* = 0.05, *p* = 0.680 |
| Mood: Average Pre-Post |  |  |
| Good-bad | ***r* = 0.25, *p* = 0.045** | *r* = 0.01, *p* = 0.918 |
| Alertness-tiredness | *r* = -0.01, *p* = 0.927 | *r* = -0.04, *p* = 0.726 |
| Calmness-restlessness | *r* = 0.16, *p* = 0.201 | *r* = -0.04, *p* = 0.739 |
| Anxiety | *r* = -0.02, *p* = 0.857 | *r* = -0.02, *p* = 0.888 |

*Notes:* LF=Low frequency, HF=High frequency, RMSSD=Root mean square of successive differences, SDNN=Standard deviation of normal-to-normal RR-intervals.

**Table S5.9.** Differences in physiological and psychological outcomes between male and female participants

|  | Male  *M* (*SD*) | Female  *M* (*SD*) | *F* | *p* | η^2^ |
| --- | --- | --- | --- | --- | --- |
| Baseline condition |  |  |  |  |  |
| Heart rate | 83.2 (11.6) | 88.4 (11.0) | 3.46 | 0.067 | 0.051 |
| Heart rate variability |  |  |  |  |  |
| LF power (ms^2^) | 2311.2 (1291.4) | 1645.3 (1038.9) | **5.46** | **0.023** | **0.077** |
| HF power (ms^2^) | 1010.8 (1095.8) | 743.1 (948.8) | 1.14 | 0.290 | 0.017 |
| LF/HF | 3.8 (2.0)) | 3.3 (1.5) | 0.98 | 0.326 | 0.015 |
| RMSSD (ms) | 45.3 (24.5) | 36.7 (22.0) | 2.28 | 0.136 | 0.034 |
| SDNN | 58.5 (19.9) | 48.2 (18.5) | **4.72** | **0.034** | **0.068** |
| Mood: Average Pre-Post |  |  |  |  |  |
| Good-bad | 4.4 (0.4) | 4.0 (0.5) | **14.06** | **< 0.001** | **0.178** |
| Alertness-tiredness | 4.2 (0.6) | 3.2 (0.6) | **24.98** | **< 0.001** | **0.278** |
| Calmness-restlessness | 4.2 (0.6) | 3.7 (0.7) | **7.34** | **0.009** | **0.102** |
| Anxiety | 9.7 (2.3) | 10.9 (2.5) | 3.83 | 0.055 | 0.056 |
|  |  |  |  |  |  |
| Stress condition |  |  |  |  |  |
| Heart rate | 91.4 (12.2) | 96.2 (14.3) | 2.07 | 0.155 | 0.031 |
| Heart rate variability |  |  |  |  |  |
| LF power (ms^2^) | 1486.9 (976.3) | 1170.0 (952.4) | 1.77 | 0.188 | 0.026 |
| HF power (ms^2^) | 623.6 (1123.7) | 628.3 (987.3) | 0.00 | 0.986 | 0.000 |
| LF/HF | 4.2 (2.1) | 3.4 (1.5) | 3.28 | 0.075 | 0.048 |
| RMSSD (ms) | 32.6 (21.2) | 29.8 (22.3) | 0.27 | 0.608 | 0.004 |
| SDNN | 45.2 (17.9) | 40.3 (19.4) | 1.08 | 0.302 | 0.016 |
| Mood: Average Pre-Post |  |  |  |  |  |
| Good-bad | 4.0 (0.5) | 3.4 (0.9) | **12.36** | **< 0.001** | **0.160** |
| Alertness-tiredness | 3.8 (0.7) | 3.0 (0.8) | **18.91** | **< 0.001** | **0.225** |
| Calmness-restlessness | 3.7 (0.6) | 3.0 (0.8) | **12.13** | **< 0.001** | **0.157** |
| Anxiety | 11.6 (2.2) | 15.2 (3.6) | **21.94** | **< 0.001** | **0.252** |

*Notes:* LF=Low frequency, HF=High frequency, RMSSD=Root mean square of successive differences, SDNN=Standard deviation of normal-to-normal RR-intervals.

**Table S5.10.** Differences in physiological and psychological outcomes between student with Swiss vs. foreign nationality

|  | Swiss  *M* (*SD*) | Other  *M* (*SD*) | *F* | *p* | η^2^ |
| --- | --- | --- | --- | --- | --- |
| Baseline condition |  |  |  |  |  |
| Heart rate | 86.0 (10.5) | 86.8 (14.1) | 0.06 | 0.803 | 0.001 |
| Heart rate variability |  |  |  |  |  |
| LF power (ms^2^) | 1988.2 (1177.1) | 1747.8 (1236.5) | 0.53 | 0.467 | 0.008 |
| HF power (ms^2^) | 919.7 (1082.3) | 678.6 (797.5) | 0.74 | 0.392 | 0.011 |
| LF/HF | 3.3 (1.6) | 4.0 (1.9) | 2.12 | 0.151 | 0.032 |
| RMSSD (ms) | 41.6 (23.7) | 36.8 (22.4) | 0.55 | 0.460 | 0.008 |
| SDNN | 53.9 (20.0) | 48.9 (18.7) | 0.83 | 0.366 | 0.013 |
| Mood: Average Pre-Post |  |  |  |  |  |
| Good-bad | 4.2 (0.5) | 4.2 (0.6) | 0.35 | 0.558 | 0.005 |
| Alertness-tiredness | 3.6 (0.7) | 3.5 (0.8) | 0.03 | 0.862 | 0.000 |
| Calmness-restlessness | 3.9 (0.7) | 4.0 (0.7) | 0.62 | 0.435 | 0.009 |
| Anxiety | 10.5 (2.4) | 9.9 (2.6) | 0.80 | 0.374 | 0.012 |
|  |  |  |  |  |  |
| Stress condition |  |  |  |  |  |
| Heart rate | 93.6 (12.3) | 65.7 (16.8) | 0.32 | 0.576 | 0.005 |
| Heart rate variability |  |  |  |  |  |
| LF power (ms^2^) | 1361.3 (1003.2) | 1141.4 (871.1) | 0.68 | 0.414 | 0.010 |
| HF power (ms^2^) | 689.9 (1120.8) | 453.5 (771.1) | 0.68 | 0.413 | 0.010 |
| LF/HF | 3.6 (1.9) | 3.9 (1.6) | 0.28 | 0.601 | 0.004 |
| RMSSD (ms) | 32.2 (22.6) | 27.7 (19.5) | 0.57 | 0.452 | 0.009 |
| SDNN | 43.7 (19.6) | 38.7 (16.6) | 0.91 | 0.343 | 0.014 |
| Mood: Average Pre-Post |  |  |  |  |  |
| Good-bad | 3.6 (0.8) | 3.7 (0.9) | 0.03 | 0.854 | 0.001 |
| Alertness-tiredness | 3.4 (0.8) | 3.1 (0.8) | 1.25 | 0.267 | 0.019 |
| Calmness-restlessness | 3.3 (0.8) | 3.3 (0.8) | 0.00 | 0.979 | 0.000 |
| Anxiety | 14.0 (3.6) | 12.9 (3.5) | 1.28 | 0.263 | 0.019 |

*Notes:* LF=Low frequency, HF=High frequency, RMSSD=Root mean square of successive differences, SDNN=Standard deviation of normal-to-normal RR-intervals.

**References**

Adul Kader, F. A. H., & Eissa, M. A. (2014). Measuring test anxiety In students aged 10-17 years in Egypt: Factor analysis and psychometric properties. *International Journal of Psycho-Educational Sciences 3*, 102–109.

Ahmed, W., Minnaert, A., Kuyper, H., & van der Werf, G. (2012). Reciprocal relationships between math self-concept and math anxiety. *Learning and Individual Differences*, *22*, 385–389. <https://doi.org/10.1016/j.lindif.2011.12.004>

Alivernini, F., Cavicchiolo, E., Manganelli, S., Chirico, A., & Lucidi, F. (2020). Students’ psychological well-being and its multilevel relationship with immigrant background, gender, socioeconomic status, achievement, and class size. *School Effectiveness and School Improvement*, *31*, 172–191. <https://doi.org/10.1080/09243453.2019.1642214>

Armstrong, N., & Welsman, J. (2019). Clarity and confusion in the development of youth aerobic fitness. *Frontiers in Physiology*, *10*, 979. <https://doi.org/10.3389/fphys.2019.00979>

Badura, P., Eriksson, C., García-Moya, I., Löfstedt, P., Melkumova, M., Sotiroska, K., Wilson, M., Brown, J., & Inchley, J. (2024). *A focus on adolescent social contexts in Europe, central Asia and Canada. Health Behaviour in School-aged Children international report from the 2021/2022 survey (Volume 7)*. WHO Regional Office for Europe.

Ding, Y., Klapp, A., & Hansen, K. Y. (2024). The importance of mathematics self-concept and self-efficacy for mathematics achievement: A comparison between public and independent schools in Sweden. *Educational Psychology*, *44*, 872–892. <https://doi.org/10.1080/01443410.2024.2410217>

Elgar, F. J., De Clercq, B., Schnohr, C. W., Bird, P., Pickett, K. E., Torsheim, T., Hofmann, F., & Currie, C. (2013). Absolute and relative family affluence and psychosomatic symptoms in adolescents (vol 91, pg 25, 2013). *Social Science & Medicine*, *94*, 129–129. <https://doi.org/10.1016/j.socscimed.2013.07.005>

Garcia-Moya, I., Paniagua, C., & Jimenez-Iglesias, A. (2025). Gender differences in adolescent school stress: A mixed-method study. *Jorurnal of Research in Adolescence*, *35*, e13057. <https://doi.org/10.1111/jora.13057>

Gilman, R., Laughlin, J. E., & Huebner, E. S. (1999). Validation of the Self-Description Questionnaire-II with an American sample. *School Psychology International*, *20*, 300–307.

Guerin, F., Marsh, H. W., & Famose, J. P. (2003). Construct validation of the Self-Description Questionnaire II with a French sample. *European Journal of Psychological Assessment*, *19*, 142–150.

Haugan, T., Muggleton, S., & Myhr, A. (2021). Psychological distress in late adolescence: The role of inequalities in family affluence and municipal socioeconomic characteristics in Norway. *PLoS One*, *16*, e0254033.

Hinz, A., Daig, I., Petrowski, K., & Brahler, E. (2012). Die Stimmung in der deutschen Bevolkerung: Referenzwerte fur den Mehrdimensionalen Befindlichkeitsfragebogen MDBF [Mood in the German population: norms of the Multidimensional Mood Questionnaire MDBF]. *Psychotherapie, Psychosomatik, Medizinische Psychology*, *62*, 52–57.

Högberg, B. (2021). Educational stressors and secular trends in school stress and mental health problems in adolescents. *Social Science and Medicine*, *270*, 113616. <https://doi.org/10.1016/j.socscimed.2020.113616>

Huan, V. S., Yeo, L. S., Ang, R. P., & Chong, W. H. (2008). The impact of adolescent concerns on their academic stress. *Educational Review*, *60*, 169–178. <https://doi.org/10.1080/00131910801934045>

Kaczmarek, M., & Trambacz-Oleszak, S. (2021). School-related stressors and the intensity of perceived stress experienced by adolescents in Poland. *International Journal of Environmental Research and Public Health*, *18*, 11791. <https://doi.org/10.3390/ijerph182211791>

Kanellopoulou, A., Vassou, C., Kornilaki, E. N., Notara, V., Antonogeorgos, G., Rojas-Gil, A. P., Lagiou, A., Yannakoulia, M., & Panagiotakos, D. B. (2022). The association between stress and children's weight status: a school-based, epidemiological study. *Children*, *9*, 1066. <https://doi.org/10.3390/children9071066>

Klinger, D. A., Freeman, J. G., Bilz, L., Liiv, K., Ramelow, D., Sebok, S. S., Samdal, O., Dür, W., & Rasmussen, M. (2015). Cross-national trends in perceived school pressure by gender and age from 1994 to 2010. *European Journal of Public Health*, *25*, 51–56. <https://doi.org/10.1093/eurpub/ckv027>

Lang, J. J. (2018). Exploring the utility of cardiorespiratory fitness as a population health surveillance indicator for children and youth: An international analysis of results from the 20-m shuttle run test. *Applied Physiology, Nutrition, and Metabolism*, *43*, 211.

Lang, J. J., Belanger, K., Poitras, V., Janssen, I., Tomkinson, G. R., & Tremblay, M. (2017). Systematic review of the relationship between 20 m shuttle run performance and health indicators among children and youth. *Journal of Science and Medicine in Sport*, doi: 10.1016/j.jsams.2017.1008.1002.

Lang, J. J., Tomkinson, G. R., Janssen, I., Ruiz, J. R., Ortega, F. B., Léger, L., & Tremblay, M. S. (2018). Making a case for cardiorespiratory fitness surveillance among children and youth. *Exercise and Sport Sciences Reviews*, *46*, 66–75.

Lang, J. J., Wolfe Philips, E., Orpana, H. M., Tremblay, M. S., Ross, R., Ortega, F. B., Silva, D. A. S., & Tomkinson, G. R. (2018). Field-based measurement of cardiorespiratory fitness to evaluate physical activity. *Bulletin of the World Health Organization*, *96*, 794–796.

Laux, L., Glanzmann, P., Schaffner, P., & Spielberger, C. D. (1981). *Das State-Trait-Angst-Inventar: Theoretische Grundlagen und Handweisung [The State-Trait-Anxiety Inventory: Theoretical foundations and manual]*. Beltz.

Leger, L. A., Mercier, D., Gadoury, C., & Lambert, J. (1988). The multistage 20 metre shuttle run test for aerobic fitness. *Journal of Sports Sciences*, *6*, 93–101.

Marsh, H. W. (1990a). A multidimensional, hierarchical self-concept: Theoretical and empirical justification. *Educational Psychology Review*, *2*, 77–172.

Marsh, H. W. (1990b). *Self-Description Questionnaire (SDQ). Manual*. University of Western Sydney at McArthur.

Marsh, H. W., Ellis, L. A., Parada, R. H., Richards, G., & Heubeck, B. G. (2005). A short version of the Self Description Questionnaire II: Operationalizing criteria for short-form evaluation with new applications of confirmatory factor analyses. *Psychological Assessment*, *17*, 81–102.

Mayorga-Vega, D., Aguilar-Soto, P., & Viciana, J. (2015). Criterion-related validity of the 20-m shuttle run test for estimating cardiorespiratory fitness: a meta-analysis. *Journal of Sport Science and Medicine*, *14*, 536–547.

Nilsson, S., Buchholz, M., & Thunberg, M. (2012). Assessing children's anxiety using the modified short State-Trait Anxiety Inventory and talking mats: A pilot study. *Nursing: Research and Practice*, *2012*, 932570.

Ortega, F. B., Zhang, K., Cadenas-Sanchez, C., Tremblay, M. S., Jurak, G., Tomkinson, G. R., Ruiz, J. R., Keller, K., Nyström, C. D., Sacheck, J. M., Pate, R., Weston, K. L., Kidokoro, T., Poon, E. T., Wachira, L.-J. M., Ssenyonga, R., Gomes, T. N. Q. F., Cristi-Montero, C., Fraser, B. J.,…Liu, Y. (2025). The Youth Fitness International Test (YFIT) battery for monitoring and surveillance among children and adolescents: A modified Delphi consensus project with 169 experts from 50 countries and territories. *Journal of Sport and Health Science*, *14*, 101012. <https://doi.org/https://doi.org/10.1016/j.jshs.2024.101012>

Putwain, D. W., Gallard, D., Beaumont, J., Loderer, K., & von der Embse, N. P. (2021). Does test anxiety predispose poor school-related wellbeing and enhanced risk of emotional disorders? *Cognitive Therapy and Research*, *45*, 1150–1162. <https://doi.org/10.1007/s10608-021-10220-w>

Ross, R., Blair, S. N., Arena, R., Church, T. S., Després, J. P., Franklin, B. A., Haskell, W. L., Kaminsky, L. A., Levine, B. D., Lavie, C. J., Myers, J., Niebauer, J., Sallis, R., Sawada, S. S., Sui, X. M., Wisloff, U., Hlth, C. L. C., Cardiology, C. C., Prevention, C. E.,…Council, S. (2016). Importance of assessing cardiorespiratory fitness in clinical practice: a case for fitness as a clinical fital sign. A scientific statement from the American Heart Association. *Circulation*, *134*, E653–E699. <https://doi.org/10.1161/Cir.0000000000000461>

Ruiz, J. R., Castro-Pinero, J., Artero, E. G., Ortega, F. B., Sjöström, M., Suni, J., & Castillo, M. J. (2009). Predictive validity of health-related fitness in youth: A systematic review. *British Journal of Sports Medicine*, *43*, 909–923.

Schelleman-Offermans, K., Vieno, A., Stevens, G. W. J. M., & Kuntsche, E. (2022). Family affluence as a protective or risk factor for adolescent drunkenness in different countries and the role drinking motives play. *Social Science & Medicine*, *311*, 115302. <https://doi.org/10.1016/j.socscimed.2022.115302>

Schwenkmezger, P., Hodapp, V., & Spielberger, C. D. (1992). *Das State-Trait-Ärgerausdrucksinventar STAXI: Handbuch [State-Trait-Anger Inventory STAXI: User manual]*. Huber.

Shoahosseini, R., & Baghaei, T. (2020). Validation of the Persian translation of the Children’s Test Anxiety Scale: A multidimensional Rasch model analysis. *European Journal of Investigation in Health, Psychology and Education*, *10*, 59–69.

Spielberger, C. D., Gorsuch, R. L., & Lushene, R. (1970). *Manual for the State-Trait-Anxiety-Inventory: STAI*. Consulting Psychologist Press.

Steyer, R., Schwenkmezger, P., Notz, P., & Eid, M. (1997). *Der Mehrdimensionale Befinlichkeitsfragebogen MDBF (Mood Questionnaire): Handanweisungen*. Hogrefe.

Thomas, C. L., & Cassady, J. C. (2021). Validation of the state version of the State-Trait Anxiety Inventory in a university sample. *Sage Open*, *11*, <https://doi.org/10.1177/21582440211031900>.

Timmerman, H. L., Toll, S. W. M., & van Luit, J. E. H. (2017). The relation between math self-concept, test and math anxiety, achievement motivation, and math achievement in 12- to 14-year-old adolescents. *Psychology, Society, & Education*, *9*, 89–103.

Torrano, R., Ortigosa, J. M., Riquelme, A., Mendez, F. J., & Lopez-Pina, J. A. (2020). Test anxiety in adolescent students: different responses according to the components of anxiety as a function of sociodemographic and academic variables. *Frontiers in Psychology*, *11*, 612270. <https://doi.org/10.3389/fpsyg.2020.612270>

Torsheim, T., Cavallo, F., Levin, K. A., Schnohr, C., Mazur, J., Niclasen, B., & Currie, C. (2016). Psychometric validation of the revised Family Affluence Scale: A latent variable approach. *Child Indicators Research*, *9*, 771–784. <https://doi.org/10.1007/s12187-015-9339-x>

Torsheim, T., Cavallo, F., Levin, K. A., Schnohr, C., Mazur, J., Niclasen, B., Currie, C., & Grp, F. D. S. (2016). Psychometric validation of the revised family affluence scale: a latent variable approach. *Child Indicators Research*, *9*, 771–784. <https://doi.org/10.1007/s12187-015-9339-x>

Vogelaar, S., Miers, A. C., Saab, N., van Loon, A. W. G., Creemers, H. E., Asscher, J. J., & Westenberg, P. M. (2024). Self-reported stressors in early adolescence: the role of educational track and ethnic background. *Journal of Early Adolescence*, *44*, 405–428. <https://doi.org/10.1177/02724316231182297>

Wren, D. G., & Benson, J. (2004). Measuring test anxiety in children: Scale development and internal construct validation. *Anxiety, Stress, & Coping*, *17*, 227–240. <https://doi.org/10.1080/10615800412331292606>

Yusefzadeh, H., Amirzadeh Iranagh, J., & Nabilou, B. (2019). The effect of study preparation on test anxiety and performance: a quasi-experimental study. *Advances in Medical Education and Practice*, *10*, 245–251. <https://doi.org/10.2147/amep.S192053>

Zheng, G., Zhang, Q. Z., & Ran, G. M. (2023). The association between academic stress and test anxiety in college students: The mediating role of regulatory emotional self-efficacy and the moderating role of parental expectations. *Frontiers in Psychology*, *14*, 1008679. <https://doi.org/10.3389/fpsyg.2023.1008679>
